# Which is the optimal antiobesity agent for patients with nonalcoholic fatty liver disease?

**DOI:** 10.3389/fendo.2022.984041

**Published:** 2022-09-02

**Authors:** Alexandra Tsankof, Georgios Neokosmidis, Evgenia Koureta, Stavroula Veneti, Evangelos Cholongitas, Konstantinos Tziomalos

**Affiliations:** ^1^ First Propedeutic Department of Internal Medicine, First Department of Internal Medicine, Medical School, Aristotle University of Thessaloniki, AHEPA Hospital, Thessaloniki, Greece; ^2^ Laiko General Hospital, Medical School, National and Kapodistrian University of Athens, Athens, Greece

**Keywords:** nonalcoholic fatty liver disease, obesity, orlistat, liraglutide, semaglutide, lorcaserin, naltrexone/bupropion, phentermine/topiramate

## Abstract

Nonalcoholic fatty liver disease (NAFLD) is the commonest chronic liver disease and affects a considerable proportion of the general population worldwide. Obesity is a major risk factor for development and progression of NAFLD and weight loss is an effective intervention for the management of NAFLD. However, few patients achieve substantial and sustained weight loss with lifestyle measures. Therefore, antiobesity agents are frequently considered in patients with NAFLD but there are limited data on their safety and efficacy. In the present review, we discuss the role of antiobesity agents in the management of NAFLD. All approved antiobesity agents appear to reduce transaminase levels and to improve steatosis in patients with NAFLD. However, their effects on fibrosis are less well studied and whether they affect liver-related outcomes, including progression to cirrhosis and hepatocellular cancer, is unknown. The glucagon-like peptide-1 receptor agonists, liraglutide and semaglutide, appear to represent a first-line option in obese patients with NAFLD and type 2 diabetes mellitus (T2DM) since they induce considerable weight loss and have been extensively studied in patients with T2DM. However, more studies are needed to evaluated their effects on liver-related and cardiovascular outcomes in patients with NAFLD, particularly in those without T2DM.

## Introduction

Nonalcoholic fatty liver disease (NAFLD) is one of the most common causes of chronic liver disease. It is estimated that about 25% of the world population has NAFLD. In the United States, it is predicted that NAFLD will affect 100 million subjects by 2030. Lately, the disease is also being observed in an increased percentage of adolescents, with an estimated prevalence of 18% (1).

Obesity is a major risk factor for both hepatic steatosis and nonalcoholic steatohepatitis (NASH) (2, 3). Importantly, even metabolically healthy obese individuals are frequently affected by NAFLD (3, 4). Given that metabolic syndrome and NAFLD share many pathogenetic pathways, particularly obesity and insulin resistance (IR), it has recently been proposed to rename NAFLD as metabolic-associated fatty liver disease (MAFLD) (5). Therefore, lifestyle modification is the first line of treatment for patients with NAFLD and consists of calorie-restricted diet and exercise (6). It has been shown that weight loss > 10% results in resolution of NASH in the majority of patients (7). However, very few patients achieve this degree of weight loss (7). Therefore, pharmacotherapy is frequently considered in these patients but relevant data are limited.

In the present review, we discuss the safety and efficacy of the available antiobesity agents in patients with NAFLD. All reviewed agents are approved for the management of patients with a body mass index (BMI) ≥ 30 kg/m^2^ and patients with BMI 27-30 kg/m^2^ and at least one obesity-related comorbidity, including hypertension, diabetes mellitus (DM), dyslipidemia, obstructive sleep apnea and established cardiovascular disease.

## Orlistat

Orlistat is a gastrointestinal reversible lipase inhibitor. Orlistat forms a covalent bond with the active serine residue site of gastric and pancreatic lipases resulting in inhibition of hydrolysis of dietary fats into absorbable free fatty acids and monoglycerides. Orlistat reduces dietary fat absorption by approximately 30% (8). Weight loss is observed within 2 weeks of use and the mean weight loss at six months is approximately 2.8 kg greater than placebo (9). The main side effects of orlistat are abdominal pain, flatulence and faecal urgency (9). Notably, cases of fulminant hepatic injury have been rarely reported in patients treated with orlistat (10).

Several studies evaluated the role of orlistat in the management of overweight or obese patients with NAFLD. In a pilot, uncontrolled study in 10 obese patients with biopsy-proven NASH, orlistat improved steatosis in 6 patients and fibrosis in 3 patients and also reduced transaminase levels (11). In another uncontrolled study in 14 patients with NASH, orlistat reduced steatosis in 10 patients, inflammation in 11 patients and fibrosis in 10 patients as evaluated in a repeat liver biopsy after 6 months of treatment (12). The severity of IR and transaminase levels also decreased (12). In a larger uncontrolled study (n = 77), treatment with orlistat for 16 weeks improved steatosis evaluated with ultrasound (13). A randomized, placebo-controlled study in 52 patients with NAFLD also reported a reduction in steatosis evaluated with ultrasound and in transaminase levels in the orlistat group (14). Another placebo-controlled study (n = 50) showed that orlistat reduces transaminase levels and has a favorable effect on the NAFLD fibrosis score and the FIB-4 score, which are established non-invasive markers of hepatic fibrosis (15). In contrast, in a randomized trial in overweight subjects with biopsy-proven NASH, a 1.400 Kcal/day diet plus vitamin E 800 IU daily was administered with or without orlistat for 36 weeks (16). Changes in weight and in transaminase levels were similar in the 2 groups (16). In addition to the weight loss-related benefit, orlistat also exerts anti-inflammatory and anti-oxidant effects that might also play a role in the improvement in liver histology and biochemistry (17).

## Liraglutide

Liraglutide is a glucagon-like peptide-1 (GLP-1) receptor agonist that induces weight loss by reducing appetite and energy intake (18). Liraglutide reduces body weight by approximately 5.6 kg more than placebo (19). The commonest side effects of liraglutide are nausea, vomiting, constipation and diarrhea (19).

In a pilot, uncontrolled, 24-week study in 19 patients with biopsy-proven NASH, liraglutide reduced transaminase levels. In addition, hepatic inflammation improved in 6 patients among 10 patients who received liraglutide for 96 weeks and underwent a repeat liver biopsy at the end of treatment (20). In another uncontrolled study in 55 Japanese patients with NAFLD diagnosed with ultrasound, liraglutide 0.9 mg/day, i.e. the approved dose in Japan, reduced transaminase levels and improved the FIB-4 score after 24 weeks. These effects were independent of weight loss (21). In a randomized, double-blind study in 82 women with NAFLD and a history of gestational diabetes mellitus (DM), treatment with liraglutide 1.8 mg daily for 1 year resulted in greater reductions in steatosis than placebo (22). In contrast, in a randomized study in 30 obese adults with NAFLD, liraglutide 3 mg/day administered for 26 weeks induced similar reductions compared with a supervised program of energy restriction plus moderate-intensity exercise in transaminase levels, steatosis evaluated with magnetic resonance imaging and in caspase-cleaved cytokeratin-18 levels, a marker of hepatocellular apoptosis (23).

A number of studies evaluated the effects of liraglutide in patients with both NAFLD and type 2 DM. In an uncontrolled study (n = 68), treatment with liraglutide 1.2 mg/day for 6 months reduced body weight, visceral fat, transaminase levels as well as liver fat content measured with magnetic resonance spectroscopy (24). The reduction in steatosis correlated with the reduction in body weight (24). In a retrospective study in 82 patients who were treated with liraglutide, sitagliptin or pioglitazone, all antidiabetic agents reduced transaminase levels but only liraglutide and pioglitazone lowered the AST to platelet ratio, a marker of hepatic fibrosis (25). Moreover, only liraglutide induced weight loss whereas semaglutide had no effect and weight increased in patients treated with pioglitazone (25). In another controlled, 24-week study in 85 patients, liraglutide induced similar reductions in transaminase levels as gliclazide and metformin (26). In a more recent study, 96 patients were randomized to receive insulin glargine, liraglutide or placebo for 26 weeks. Liraglutide reduced body weight, visceral adipose tissue, waist circumference as well as steatosis assessed with magnetic resonance spectroscopy whereas insulin had no effect (27). In a larger, randomized, 12-week study (n = 127), liraglutide 0.6-1.2 mg/day reduced body weight and transaminase levels more than metformin (28). In a randomized, 26-week study (n = 75), liraglutide 1.8 mg/day reduced steatosis assessed with magnetic resonance imaging and visceral adipose fat more than insulin glargine and to a similar degree with sitagliptin (29). In addition to the weight loss-related benefit, liraglutide also exerts anti-inflammatory and anti-oxidant effects that might also play a role in the improvement in liver histology and biochemistry (30).

## Semaglutide

Semaglutide is a novel GLP-1 receptor agonist that appears to induce greater weight loss than liraglutide and has the additional advantage that it is administered once weekly, which might improve adherence to treatment (31). Moreover, the pharmacokinetic characteristics of semaglutide are not affected in patients with cirrhosis attributable to parenchymal liver disease, including NAFLD (32). Similarly to liraglutide and other GLP-1 receptor agonists, the commonest side effects of liraglutide are nausea, vomiting, constipation and diarrhea (33).

Emerging data suggest that semaglutide might be useful in patients with NAFLD. In a retrospective cohort study in 43 patients with type 2 DM, treatment with semaglutide for 6 months reduced transaminase levels (34). In a *post-hoc* analysis of 2 randomized, double-blind study in obese patients with or without type 2 DM, treatment with semaglutide 0.5-1.0 mg/week for 52-102 weeks also reduced transaminase levels, which were normalized in 25-46% of the patients (35). These effects were primarily due to weight loss (35). In a 72-week, randomized, placebo-controlled study in 320 patients with biopsy proven NASH, once-daily semaglutide at a dose of 0.1, 0.2 or 0.4 mg induced NASH resolution (defined as no more than mild residual inflammatory cells and no hepatocyte ballooning) without worsening of fibrosis (defined as an increase of one stage or more on the Kleiner fibrosis classification scale) in 40, 36 and 59% of the patients compared with 17% in the placebo group (p < 0.001 for semaglutide 0.4 mg vs. placebo) (36). However, the change in fibrosis stage with no worsening of NASH did not differ between the semaglutide and placebo groups (36). Notably, the mean weight loss was 13% in the 0.4-mg group vs. 1% in the placebo group (36). In another randomized, double-blind, placebo-controlled, 72-week study in 67 patients with NAFLD, semaglutide 0.4 mg/day reduced steatosis evaluated with MRI and transaminase levels as well as visceral abdominal fat and systolic blood pressure but had no effect on liver stiffness evaluated with magnetic resonance elastography (37). Semaglutide might be more effective when combined with other agents that are being evaluated in patients with NAFLD. In a randomized, 24-week, phase 2a study, semaglutide 2.4 mg once weekly in combination with the acetylcoenzyme A carboxylase inhibitor firsocostat, and/or the farnesoid X receptor agonist cilofexor was more effective than semaglutide monotherapy in improving a score incorporating liver stiffness (measured with vibration-controlled transient elastography), liver steatosis (assessed with the controlled attenuation parameter) and AST levels in 108 patients with NASH and mild to moderate fibrosis (38). In addition to the weight loss-related benefit, semaglutide also exerts anti-inflammatory effects, reduces lipogenesis, increases beta-oxidation and endoplasmic reticulum stress, which might also play a role in the improvement in liver histology and biochemistry (39).

## Lorcaserin

Lorcaserin is a selective 5-HT2C agonist, which acts by stimulating the 5-HT2C receptors of the pro-opiomelanocortin (POMC) neurons in the arcuate nucleus, inducing the release of α-melanocortin-stimulating hormone, which in turn acts on melanocortin-4 receptors in the paraventricular nucleus to suppress appetite (8, 40). The average weight loss induced by lorcaserin at the dose of 10 mg twice daily is approximately 3.6 kg greater than placebo (41). The most frequent side effects of lorcaserin are headache and dizziness (41). However, there are very limited data on the effects of lorcaserine in patients with NAFLD. In a randomized, placebo-controlled, 6-month study in 48 obese patients, lorcaserine improved the fatty liver index, a marker of steatosis (42). Notably, this benefit was independent of weight loss (42). Lorcaserine has been shown to exert anti-inflammatory effects, which might contribute to the reduction in hepatic steatosis, in addition to the weight loss-related benefit (41).

## Naltrexone/bupropion

The combination of naltrexone, a non-selective opioid receptor antagonist, and buproprion, an inhibitor of dopamine and norepinephrine transporters, at the dose of 16/180 mg twice daily, induces weight loss approximately 4.1% greater than placebo (43). The most frequent side effects of naltrexone/bupropion are nausea, headache and constipation (43). Naltrexone/bupropion might increase blood pressure and is contraindicated in patients with uncontrolled hypertension, which is of concern given the high cardiovascular risk in patients with NAFLD (43). In a *post-hoc* analysis of 4 randomized, 56-week studies in 2073 obese patients, naltrexone/bupropion reduced transaminase levels and the FIB-4 index and this effect was more pronounced in patients with elevated transaminase levels (44). Naltrexone/bupropion was also shown to reduce high-sensitivity C-reactive protein levels and this anti-inflammatory effect might contribute to its potentially beneficial effect of NAFLD progression in addition to the the weight loss-related benefit (43).

## Phentermine/topiramate

The combination of phentermine, a sympathomimetic amine anorectic that stimulates the release of norepinephrine and epinephrine, and phentermine, an inhibitor of the norepinephrine transporter that suppresses appetite by activation of POMC arcuate nucleus neurons, at the dose of 15/92 mg/day, induces weight loss approximately 8.8 kg greater than placebo (45). The commonest side effects of phentermine/topiramate are dry mouth, paresthesia and constipation (45). Pulmonary hypertension is the most serious adverse event of this agent but has been rarely observed in clinical trials (45). Even though phentermine/topiramate appears to induce greater weight loss than other antiobesity agents, it has not evaluated in patients with NAFLD. However, in patients with moderate hepatic impairment, the maximum recommended dose is 7.5/46 mg per day (46). Similar to other antiobesity agents, phentermine/topiramate was also shown to reduce hs-CRP levels (45). Moreover, an increase in the insulin-sensitizing adipokine adiponectin was observed in patients treated with this agent (45). However, it is unclear if these improvements will translate into benefits in liver histology or biochemistry in patients with NAFLD.

## Conclusions

Several agents are currently approved for the management of obesity. Most of them have been evaluated in patients with NAFLD and appear to reduce transaminase levels and to improve steatosis (**Table 1**). However, their effects on fibrosis are less well studied and whether they affect liver-related outcomes, including progression to cirrhosis and hepatocellular cancer, is unknown. The GLP-1 receptor agonists, liraglutide and semaglutide, appear to represent a first-line option in obese patients with NAFLD and T2DM since they induce considerable weight loss and have been extensively studied in patients with T2DM. However, more studies are needed to evaluate their effects on liver-related and cardiovascular outcomes in patients with NAFLD, particularly in those without T2DM.

**Table 1:** Main characteristics of currently approved antiobesity agents.

Agent	Mean additional weight loss compared with placebo (kg)	Effects on nonalcoholic fatty liver disease	Commonest side effects
Orlistat	2.8	↓ transaminase levels Improvement in hepatic steatosis, inflammation and fibrosis	Abdominal pain, flatulence, faecal urgency
Liraglutide	5.6	↓ transaminase levels Improvement in hepatic steatosis and fibrosis Unknown effects on hepatic inflammation	Nausea, vomiting, constipation, diarrhea
Semaglutide	6.3	↓ transaminase levels Improvement in hepatic steatosis and inflammation No effect on hepatic fibrosis	Nausea, vomiting, constipation, diarrhea
Lorcaserin	3.6	Improvement in hepatic steatosis Unknown effects on transaminase levels and on hepatic inflammation and fibrosis	Headache, dizziness
Naltrexone/bupropion	4.1	↓ transaminase levels Improvement in hepatic fibrosis Unknown effects on hepatic steatosis and inflammation	Nausea, headache, constipation
Phentermine/topiramate	8.8	Not evaluated in patients with nonalcoholic fatty liver disease	Dry mouth, paresthesia, constipation

↓, lowering.

## Author contributions

AT, GN, EK and SV wrote the first draft. EC and KT revised the draft. All authors contributed to the article and approved the submitted version.

## Conflict of interest

The authors declare that the research was conducted in the absence of any commercial or financial relationships that could be construed as a potential conflict of interest.

## Publisher’s note

All claims expressed in this article are solely those of the authors and do not necessarily represent those of their affiliated organizations, or those of the publisher, the editors and the reviewers. Any product that may be evaluated in this article, or claim that may be made by its manufacturer, is not guaranteed or endorsed by the publisher.
